# Cross-cultural adaptation and validation to Brazil of the Obesity-related Problems Scale

**DOI:** 10.1590/S1679-45082017AO4004

**Published:** 2017

**Authors:** Andreia Mara Brolezzi Brasil, Fábio Brasil, Angélica Aparecida Maurício, Regina Maria Vilela

**Affiliations:** 1 Universidade Federal do Paraná, Curitiba, PR, Brazil.

**Keywords:** Obesity, Quality of life, Translating, Psychometrics, Validation studies, Obesidade, Qualidade de vida, Tradução, Psicometria, Estudos de validação

## Abstract

**Objective:**

To validate a reliable version of the Obesity-related Problems Scale in Portuguese to use it in Brazil.

**Methods:**

The Obesity-related Problems Scale was translated and transculturally adapted. Later it was simultaneously self-applied with a 12-item version of the World Health Organization Disability Assessment Schedule 2.0 (WHODAS 2.0), to 50 obese patients and 50 non-obese individuals, and applied again to half of them after 14 days.

**Results:**

The Obesity-related Problems scale was able to differentiate obese from non-obese individuals with higher accuracy than WHODAS 2.0, correlating with this scale and with body mass index. The factor analysis determined a two-dimensional structure, which was confirmed with χ^2^/df=1.81, SRMR=0.05, and CFI=0.97. The general a coefficient was 0.90 and the inter-item intra-class correlation, in the reapplication, ranged from 0.75 to 0.87.

**Conclusion:**

The scale proved to be valid and reliable for use in the Brazilian population, without the need to exclude items.

## RESUMO

**Objetivo:**

Validar uma versão confiável da *Obesity-related Problems Scale* em português para utilização no Brasil.

**Métodos:**

A *Obesity-related Problems Scale* foi traduzida e adaptada transculturalmente. Posteriormente, foi autoaplicada simultaneamente à versão de 12 itens da *World Health Organization Disability Assessment Schedule* 2.0 (WHODAS 2.0) a 50 pacientes obesos e a 50 indivíduos não obesos, sendo reaplicada à metade deles após 14 dias.

**Resultados:**

A *Obesity-related Problems Scale* foi capaz de diferenciar obesos de não obesos com maior acurácia que a WHODAS 2.0, estando correlacionada a esta e com o índice de massa corporal. A análise fatorial determinou estrutura bidimensional, que foi confirmada com χ^2^/df=1,81, SRMR=0,05, and CFI=0,97. O coeficiente a geral foi de 0,90 e a correlação intraclasse interitem, na reaplicação, variou de 0,75 a 0,87.

**Conclusão:**

A escala provou ser válida e confiável para ser utilizada na população brasileira, sem necessidade de exclusão de itens.

## INTRODUCTION

In a 39-year analysis in 186 countries, conducted between 1975 and 2014, the prevalence of obesity in adults increased from 3.2 to 10.8%, in males, and from 6.4 to 14.9%, in females.^(^ ^1^ ^)^ The Brazilian Institute of Geography and Statistics (IBGE - *Instituto Brasileiro de Geografia e Estatística*) and the Ministry of Health have made available data sufficient for a 41-year analysis: in Brazil, between 1974 and 2015, the prevalence of adult obese men increased from 2.8 to 18.1%; in the case of adult women, this increase went from 8 to 19.7%.^(^ ^2^ ^,^ ^3^ ^)^ Since obesity is classified as a disease,^(^ ^4^ ^)^ a pandemic is therefore characterized.

The diagnosis of obesity, according to the World Health Organization (WHO), requires a body mass index (BMI) ≥30kg/m^2^; (25kg/m^2^≤ BMI ≤30kg/m^2^, and a BMI ≤18.5kg/m^2^ determine overweight and underweight, respectively).^(^ ^5^ ^)^ Many other diseases present with a direct causal relation to obesity, such as arterial hypertension, dyslipidemia, type 2 *diabetes mellitus*, coronary artery disease, stroke, biliary lithiasis, hepatic steatosis, osteoarthritis, sleep apnea, cognitive dysfunction, various types of cancer (colorectal, postmenopausal breast, endometrium, kidney, esophagus, pancreas, and liver), depression, anxiety, and chronic body pain.^(^ ^6^ ^-^ ^8^ ^)^ Additionally, obese people are constantly targets of discrimination in all sectors of society, including the job market, education, the media, and even in healthcare − which limits their opportunities with generalized negative stereotype comments that they are lazy, disheveled, and less competent,^(^ ^9^ ^)^ contributing towards a marked decrease in quality of life of this population.

Among the specific psychometric scales to evaluate the impact of obesity on health-related quality of life (HRQoL), those that stand out due to their ample international use, facilitating the conduction of multicenter comparative studies are the Obesity-related Well-Being (ORWELL 97),^(^ ^10^ ^)^ the Impact of Weight on Quality of Life (IWQOL-Lite),^(^ ^11^ ^)^ and the Obesity-related Problem Scale (OP).^(^ ^12^ ^)^ In this study, the OP was selected because it is the only one of the three that was validated in non-obese people as well, and can be used to measure the HRQoL before and after potentially curative obesity interventions, such as diets and bariatric surgery.^(^ ^13^ ^,^ ^14^ ^)^

## OBJECTIVE

To validate a reliable version of the Obesity-related Problems Scale in Portuguese for use in Brazil.

## METHODS

### Transcultural adaptation

The process of transcultural adaptation of the OP was carried out based on systematizations proposed by several authors.^(^ ^15^ ^-^ ^17^ ^)^ Two independent translations were made from English to Brazilian Portuguese; the first, by group of three dieticians fluent in English; the second, by a sworn notarized translator, all of them with Brazilian Portuguese as their native language. The translations were independently back-translated into English by two notarized translators who had English as their native language. The back-translations were paired with each other for blinding, and with the original instruments. Then a translator, with English as native language, who had not participated in the previous phase, established an equivalence of to 100% between the pairs of items.

Three Brazilian dieticians, who did not participate in the previous stages, based on the equivalence established between the items and on their professional experience, drew up, in agreement, a pretest version of OP adapted for Brazilian Portuguese.

Five obese patients were randomly selected, who were patients seen at the outpatient obesity clinic of the *Hospital de Clínicas da Universidade Federal do Paraná* (UFPR), along with five non-obese employees of UFPR, all of them older than 18 years. The individuals selected answered the pretest version of the OP, as well as their impression as to the clarity and ease of the items.

A psychiatrist, a psychologist, and a dietitian, in agreement, pondered the results of the pretest version and prepared the final version, transculturally adapted for the Brazilian population, of OP (Chart 1). Posteriorly, this version was approved in an analogous pretest, in which no participant reported difficulty in understanding or answering the items.

**Chart 1:** Brazilian version of the Obesity-related Problems Scale (OP)

*OP1. Receber amigos em casa*
*OP2. Visitar a casa de parentes ou amigos*
*OP3. Ir a restaurantes*
*OP4. Fazer atividades na comunidade (cursos etc.)*
*OP5. Passar férias fora de casa*
*OP6. Experimentar e comprar roupas*
*OP7. Banhar-se em locais públicos (praia, piscina etc.)*
*OP8. Relações íntimas (beijo, sexo etc.)*

*Os itens da OP estão representados pela sigla “OP” seguida de seu número de ordenamento. Todos eles devem ser respondidos em escala Likert da seguinte forma: (1) “Me incomoda muito”; (2) “Me incomoda mais ou menos”; (3) “Me incomoda um pouco”; e (4) “Não me incomoda”.*

### Study design and subjects

This is an observational study of quantitative nature carried out in obese and non-obese Brazilians aged over 18 years, during the period from April 28, 2015, to September 29, 2015, in the city of Curitiba (PR).

The transculturally adapted OP was self-applied concurrently with the 12-item version of the World Health Organization Disability Assessment Schedule 2.0 (WHODAS 2.0)^(^ ^18^ ^)^ − used as a validation criterion −, in a group of 50 obese patients who did follow-up at the outpatient obesity clinic of Clinics Hospital of the UFPR, and a Control Group, paired by age and sex, of 50 non-obese inhabitants of Curitiba. After 2 weeks, the OP was reapplied to half of the individuals from each group, chosen randomly, but with the proportion maintained between the sexes.

The sample selection was made by convenience, covering all the individuals who met the inclusion criteria until the number of participants needed for the research performance was complete. Excluded were pregnant women, those younger than 18 years, individuals with restrictions in their responsibilities as to autonomy, individuals with visual or hearing problems (reported or perceived), and those who did not agree to participate in the investigation or to fill out the Informed Consent Form.

### Ethical aspects

The transcultural adaptation and validation were authorized by the main author of the OP,^(^ ^12^ ^)^ as well as the dissemination of the version transculturally adapted for Brazilian Portuguese. Using ethical criteria, we chose to work with the minimum number of observations considered ideal for the statistical analysis (n=100).^(^ ^19^ ^)^

The research was conducted within the standards required by the Declaration of Helsinki and approved by the UFPR Ethics Committee with CAAE: 38627514.8.0000.0102.

### Statistical analysis

Calculation of the OP scores was done based on the simple sum of the value of each item, using the inverted Likert scale, as per the original study,^(^ ^12^ ^)^ and its posterior transformation into values between zero to 100; more elevated scores indicated a greater psychosocial commitment and worse HRQOL.

The validity of the convergent criterion was investigated by means of Spearman’s correlation coefficients of OP with WHODAS 2.0 and the BMI. It was also verified, through the Mann-Whitney U test (validity of the discriminating criterion), the capacity of the scale to differentiate the following groups: obese, non-obese, men, women, and overweight and normoweight individuals.

Seeking the validity of the construct, an exploratory factor analysis (EFA) was performed using the method of maximum likelihood with oblique matrix rotation by maximal proportions. The number of extracted factors was determined from the analysis of the point of inflection of the scree test. The items that do not present with a minimal communality of 0.4, with factor extraction, should be considered invalid.^(^ ^20^ ^)^ Ideally, elevated factor loads should be used in order to frame the items in their respective domains; for this, values over 0.71 were considered excellent.^(^ ^21^ ^)^

A confirmatory factor analysis (CFA) was performed using the maximum likelihood method. The tested models were unidimensionality, proposed by the original study,^(^ ^12^ ^)^ and item distribution, suggested by the EFA. For model fit, it was required that the χ^2^ divided by the number of degrees of freedom (χ^2^/df) be lower than 3^(^ ^22^ ^)^ and that the comparative fit index (CFI) and the standardized residual root mean square (SRMR) have a value of more than 0.95 and less than 0.08, respectively, as per recommended for samples smaller than 250 observations.^(^ ^23^ ^)^ As a complement to CFA, calculation of the internal consistency was required, with a necessary Cronbach coefficient a ≥0.7 in the instrument as a whole, and in each of the individual domains for the validity of the construct to be confirmed.^(^ ^19^ ^)^

As to reliability, calculation of reproducibility (precision) by means of the intraclass correlation coefficients (ICC), with necessary values of more than 0.7 relative to the two applications of the OP.^(^ ^19^ ^)^ Responsivity (accuracy) was verified in a comparative manner with WHODAS 2.0, by analysis of the areas under the Receiver Operating Characteristics (ROC) curves. This analysis was possible due to the meticulous pairing between the obese and non-obese individuals, which were confronted.

Calculations were made using the Statistical Package for the Social Sciences software, version 21.0, with extension Analysis of Moment Structures; the level of significance attributed was 0.05.

## RESULTS

All individuals (80% women) responded completely to the two instruments, OP and WHODAS 2.0, with no loss of data. The descriptive variables of the study population, including the OP scores, which did not show a normal distribution as per the Kolmogorov-Smirnov test, are exposed on table 1.

**Table 1:** Sociodemographic, anthropometric variables and Obesity-related Problems Scale scores of the sample population

Variable	Obese	Non-obese	Women	Men
Age*, (years)	44.48 (11.70) (18-62)	44.48 (11.70) (18-62)	45.50 (11.50) (21-62)	40.40 (11.57) (18-55)
Body mass index*, (kg/m^2^)	40.42 (5.55) (31.64-59.69)	24.05 (3.00) (18.50-29.39)	32.30 (9.73) (18.36-59.69)	31.97 (7.86) (21.67-46.28)
^†^monthly family income *per capita* ^†^ *, (Reais)*	827.48 (223.14-4462.80)	1000.00 (219.44-3750.00)	964.88 (219.44-4462.80)	1022.73 (219.44-3000.00)
Years of study^†^	11 (4-16)	11 (4-17)	11 (4-17)	11 (4-16)
OP^†^, scale from zero to inverted 100	39.58 (0.00-100.00)	4.17 (0.00-37.50)	20.83 (0.00-100.00)	6.25 (0.00-41.67)

* mean; standard deviation and minimal and maximal intervals between parentheses: μ(σ) (min-max); † median; minimal and maximal intervals between parentheses: med (min-max); n=100 (50% obese; 80% women).

OP: Obesity-related Problems Scale.

The OP scores were capable of differentiating the obese from the non-obese (U=244; z=-6.97; p<0.01), the obese from overweight individuals (U=76; z=-4.46; p<0.01), and women from men (U=433.5; z=-3.17; p<0.01); nevertheless, they were not able to differentiate overweight individuals from normoweight individuals (U=250.5; z=-0.03; p=0.97). The OP showed a Spearman correlation coefficient of 0.67 (p<0.01) with WHODAS 2.0 and 0.66 (p<0.01) with the BMI.

**Figure 1:**
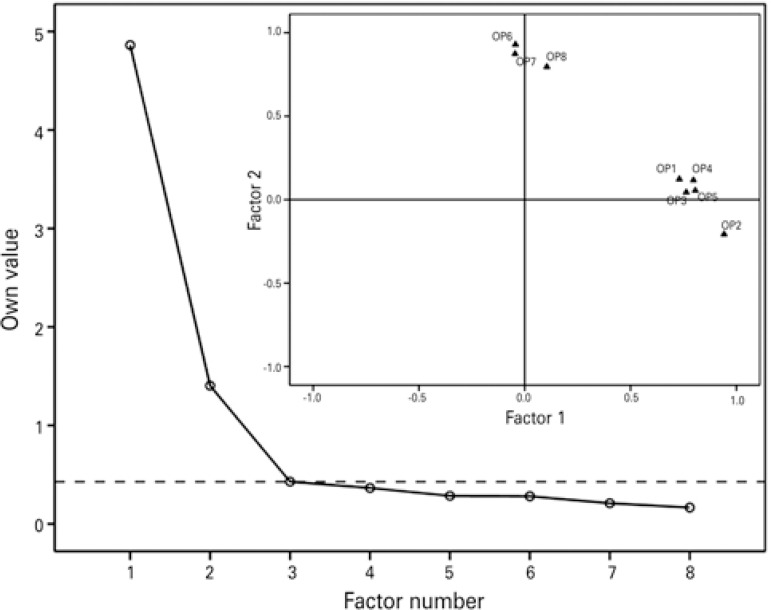
Scree graphs of the factors and of item dispersion of the Obesity-related Problems Scale. The dashed line crosses the cutoff point used to determine the number of extracted factors; n=100 (50% obese; 80% women)

The value of 0.87 was calculated for the Kaiser-Meyer-Olkin coefficient, which, associated with a sphericity test with a significance of p<0.01, determined that the database was sufficient for the execution of the EFA. The scree test presented with a well-defined inflection point, determining the extraction of two factors (Figure 1), which answered for 78.32% of the variance; the individual items exhibited satisfactory communalities and factor loads (Table 2).

**Table 2:** Exploratory factor analysis of the Obesity-related Problems Scale

Item	Communality	Factor loading	

		60.78%	17.54%
OP1	0.65	0.80*	0.54
OP2	0.71	0.82*	0.33
OP3	0.62	0.79*	0.48
OP4	0.76	0.86*	0.57
OP5	0.70	0.84*	0.51
OP6	0.82	0.49	0.91*
OP7	0.72	0.45	0.85*
OP8	0.74	0.56	0.86*

Extraction by the maximum likelihood method with promax rotation; matrix of the structures. The factors are represented by the percentage of the variance explained.

* relevant factor load; n=100 (50% obese; 80% women).

OP: Obesity-related Problems Scale.

The CFA assessed the hypothesis of a structure with single domain in OP with the following values for the adjustment indexes: χ***^2^***/df=7.70, SRMR=0.13 and CFI=0.76. They proved to not be the correct model. When the theoretical structure was tested and the items were divided into two inter-related domains (“sociability” – OP1 to OP5 – and “corporality” – OP6 to OP8 –), we identified χ^2^/df=1.81, SRMR=0.05 and CFI=0.97, validating this model for the study population.

The a coefficient calculated for the domain “sociability” was 0.91; for “corporality,” it was 0.90; and for the instrument as a whole, it was 0.90 – values sufficient to corroborate the validity of the construct.

The OP demonstrated satisfactory reproducibility between the application and the reapplication, by means of the CCI of 0.93 in reference to the total score. The CCI of the individual items varied from 0.75 to 0.87.

**Figure 2:**
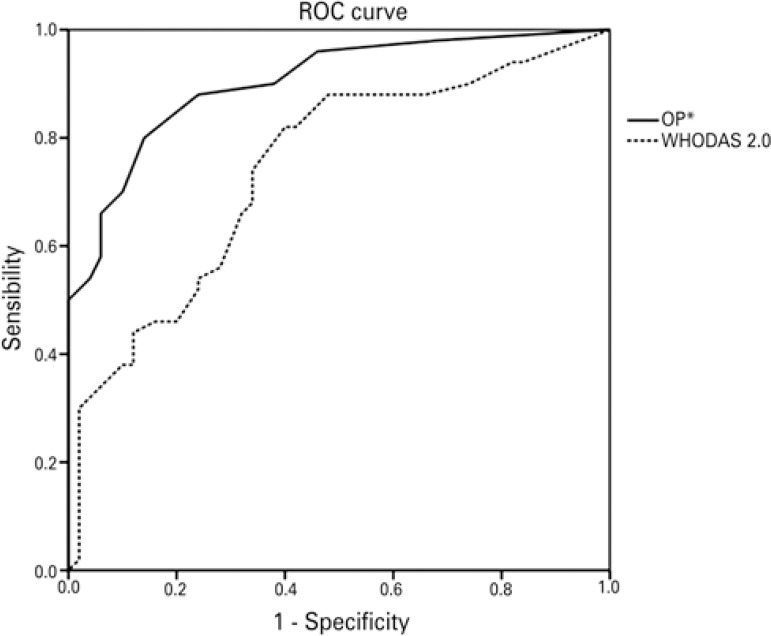
Accuracy in differentiating obese and non-obese

The area under the ROC curve comparing the group of obese patients and the group of non-obese individuals was 0.74 (p<0.01) for the WHODAS 2.0 scale, and 0.90 (p<0.01) for the OP, conferring greater accuracy to the latter (Figure 2).

* inverted scale.

ROC: Receiver Operating Characteristics; OP: Obesity-related Problems Scale; WHODAS 2.0: 12-item version of the World Health Organization Disability Assessment Schedule 2.0; n=50 (50% obese; 80% women).

## DISCUSSION

The OP instrument is a scale of outcomes reported by patients, which was developed and validated in Sweden, based on a sample of 12,296 obese and 1,017 non-obese individuals. It primarily measures the impact of excess weight on the psychosocial function;^(^ ^12^ ^)^ it has been transculturally adapted for Spain,^(^ ^24^ ^)^ South Korea,^(^ ^25^ ^)^ and Norway;^(^ ^14^ ^)^ therefore, its use is corroborated at international level and in a large population. The a coefficient, calculated as 0.90 in the present study was similar to that of the original OP instrument^(^ ^12^ ^)^ and to the adapted versions for Spain,^(^ ^24^ ^)^ South Korea,^(^ ^25^ ^)^ and Norway,^(^ ^14^ ^)^ which presented with a coefficients of 0.89-0.92, 0.93, and 0.91, respectively, suggesting that there is a general analogy in the psychic construct among the populations. Studies conducted in the abovementioned countries validated the OP on a single dimension. The present study performed the validation in a two-dimensional manner, as per the results of the CFA, which does not disqualify the validity of the general construct, but merely reflects the presence of its own characteristics in the latent psychological traces of the Brazilian population.

Considering these specific results identified by the CFA in the Brazilian population, the first domain – items OP1 to OP5 – was named “sociability”, due to the common characteristic of these items to determine latent traces related to the individual’s integration with the social group in which they live.^(^ ^26^ ^)^ A latent trace refers to an intrinsic capacity; thus, the use of the term “sociability” instead of “socialization,” which emphasizes an active process;^(^ ^27^ ^)^ or of “social behavior,” which is the product of various “social behaviors,”^(^ ^26^ ^)^ that is, various latent traces. The second domain – items OP6 to OP8 – was called “corporeality,” as it characterizes the form in which the brain uses the physical body to relate to the environment, notably, with the social milieu;^(^ ^28^ ^)^ as opposed to the term “physicality,” which is substantially relative to the body, mechanically, and has little psychic denotation.^(^ ^28^ ^)^

The significant correlation (convergent validity) with the instrument, WHODAS 2.0, which knowingly measures the HRQoL,^(^ ^18^ ^)^ verified that the instrument OP is also capable of measuring HRQoL. Furthermore, the significant correlation established with the BMI determined that the OP is endowed with specificity to measure traces associated with body weight and with obesity.^(^ ^5^ ^)^ The OP may be considered a specific psychometric scale for obesity, capable of measuring HRQOL in a Brazilian population sample.

Attempts were made to discriminate groups (discriminating validity) in order to have an indirect assessment of responsiveness.^(^ ^19^ ^)^ In this regard, OP proved capable of differentiating obese (patients) from non-obese (healthy) with greater accuracy than the WHODAS 2.0, which is a generic scale of outcomes reported by patients relative to obesity, as was expected in the theoretic proposition.^(^ ^12^ ^)^ The fact that this instrument does not distinguish people with normal weight from those who are overweight demonstrates the non-discrimination among healthy individuals, since being overweight is not classified as a disease. Additionally, it differentiated between men and women − these with worse scores, as has been systematically verified in patient-reported outcome scales,^(^ ^29^ ^,^ ^30^ ^)^ including in the original study.^(^ ^12^ ^)^ OP was also adequately reproducible, based on the assumption (and even suggesting) that clinical changes were, in fact, negligible between the two applications.

The availability for Brazil of a valid and reliable version of OP fills a gap, based on which the clinical evaluation of the obese patient is no longer restricted to anthropometric, laboratory, and bioimpedance data.^(^ ^31^ ^,^ ^32^ ^)^ Now one can approach and measure, by means of the point of view of the persons evaluated, the psychic suffering related to obesity, which is evident but not duly quantified, by the stigmatization of a prejudiced contemporary society,^(^ ^9^ ^)^ in which the search for the ideal silhouette is an obsession for many.^(^ ^33^ ^)^

Despite the recommendation of the developers of OP to consider scores lower than 40 as a mild psychosocial involvement, between 40 and 59 as moderate, and higher than 60 as severe,^(^ ^12^ ^)^ the interpretation of the scores of the outcome scales reported by patients, based on a more up-to-date view, should not be based on absolute values, but on variations of these scores when establishing an intervention or treatment, determining its efficacy.^(^ ^34^ ^,^ ^35^ ^)^ We point out that the OP presents with the advantage of having been validated as well for the non-obese, and can identify disparities in the benefits that different interventions cause in the HRQoL, even when they are effectively curative. After all, it has already been verified that former obese people differ in psychosocial aspects, according to the treatment given to lose weight.^(^ ^13^ ^,^ ^36^ ^)^

This study presents the limitation of having been restricted to the population of only one Brazilian city. However, the semantic standardization established as cultured/educated (since as the transcultural adaptation of the OP did not use regional idiomatic expressions), associated with the phenomenon of globalization, both present in Brazil,^(^ ^37^ ^,^ ^38^ ^)^ allow the results be nationally acceptable - except in isolated population groups.

Despite having been used successfully in 13 to 18 year-old adolescents,^(^ ^39^ ^)^ OP presents as an intrinsic limitation the fact of not being appropriate for the pediatric population. Additionally, contrary to the scales of ORWELL-97 and IWQOL-Lite, which also evaluated somatic functional domains, the OP instrument focuses on the psychosocial function,^(^ ^40^ ^)^ so that the joint application with a scale of patient-reported outcomes directed to measure general function, such as WHODAS 2.0, becomes imperative for a convincing evaluation of HRQoL.

## CONCLUSION

The Obesity-related Problems Scale proved to be valid and reliable for use in the Brazilian population, both in obese and in non-obese individuals. No item of the instrument needed to be excluded. The transculturally adapted version for Brazil is freely available for research or for clinical practice, and it is not necessary to notify the authors.
